# Control of Abnormal Synchronization in Neurological Disorders

**DOI:** 10.3389/fneur.2014.00268

**Published:** 2014-12-16

**Authors:** Oleksandr V. Popovych, Peter A. Tass

**Affiliations:** ^1^Institute of Neuroscience and Medicine – Neuromodulation, Jülich Research Center, Jülich, Germany; ^2^Department of Neurosurgery, Stanford University, Stanford, CA, USA; ^3^Department of Neuromodulation, University of Cologne, Cologne, Germany

**Keywords:** neuronal synchronization, electrical stimulation, sensory stimulation, spike timing-dependent plasticity, anti-kindling, coordinated reset neuromodulation, noise stimulation

## Abstract

In the nervous system, synchronization processes play an important role, e.g., in the context of information processing and motor control. However, pathological, excessive synchronization may strongly impair brain function and is a hallmark of several neurological disorders. This focused review addresses the question of how an abnormal neuronal synchronization can specifically be counteracted by invasive and non-invasive brain stimulation as, for instance, by deep brain stimulation for the treatment of Parkinson’s disease, or by acoustic stimulation for the treatment of tinnitus. On the example of coordinated reset (CR) neuromodulation, we illustrate how insights into the dynamics of complex systems contribute to successful model-based approaches, which use methods from synergetics, non-linear dynamics, and statistical physics, for the development of novel therapies for normalization of brain function and synaptic connectivity. Based on the intrinsic multistability of the neuronal populations induced by spike timing-dependent plasticity (STDP), CR neuromodulation utilizes the mutual interdependence between synaptic connectivity and dynamics of the neuronal networks in order to restore more physiological patterns of connectivity via desynchronization of neuronal activity. The very goal is to shift the neuronal population by stimulation from an abnormally coupled and synchronized state to a desynchronized regime with normalized synaptic connectivity, which significantly outlasts the stimulation cessation, so that long-lasting therapeutic effects can be achieved.

## 1 Introduction

Several brain disorders, such as Parkinson’s disease (PD), essential tremor, epilepsy, and tinnitus are characterized by abnormally strong, pathological neuronal synchronization (1–10). In PD, for example, excessive synchronization is associated with motor impairment (2, 7, 11–15), whereas neurons exhibit an asynchronous firing under physiological conditions (2, 16). Reduction of synchronized oscillations in β-band frequencies (8−35 Hz) by medication with dopaminergic drugs is positively correlated with amelioration of motor symptoms in PD patients (7, 13, 14). On the other hand, low-frequency (5−20 Hz) stimulation of subthalamic nucleus (STN) or its afferent fibers, which is intended to boost synchronous oscillations at these frequencies, results in a significant clinical deterioration of PD symptoms and motor functions in human patients and parkinsonian rodents (15, 17–22).

In tinnitus, abnormal patterns of enhanced synchronized neuronal activity were observed in human patients and animal models in brain areas differentiated from peripheral input (5, 6, 10, 23–27). In particular, pathologically elevated and persistent neuronal rhythms at δ-band (1.5−4 Hz) as well as at γ-band (>30 Hz) frequencies were found in patients with chronic tinnitus as compared to healthy controls (5, 6, 26–28), which correlate with tinnitus-related distress and tinnitus loudness. Furthermore, a positive association was found between normalization of this neuronal activity and reduction of tinnitus severity (25, 27, 29). Taken together, abnormal neuronal synchronization can significantly impair neural processes and brain functions and serves as target for novel therapies.

Nowadays, the standard therapy for medically refractory PD patients is electrical high-frequency (HF) deep brain stimulation (DBS) (30). For this, an electrical HF (>100 Hz) pulse train is delivered to a target brain area through a depth electrode. The treatment of severe neurological and psychiatric diseases such as PD, tremor, dystonia, chronic and phantom pain, major depression, obsessive–compulsive disorder, Tourette syndrome, and epilepsy with DBS is a rapidly growing and promising field (7, 30–32). The mechanism of HF DBS is, as yet, not fully understood. One may distinguish between local and non-local effects of HF stimulation. Locally, in the vicinity of the stimulation electrode, axons rather than cell bodies (somas) get activated (21, 33), while the latter can even be effectively inhibited by HF stimulation (34–36) via, e.g., synaptic inhibition or depolarization blockade (37–39). The stimulation-induced axonal activity propagates antidromically and orthodromically (21, 40) and can change the firing in the output structures downstream to the neuronal target population. In particular, the globus pallidum interior (GPi) might strongly be involved in the mechanisms of DBS (41–43). HF stimulation of the STN regularizes GPi firing (41), and this restores the responsiveness of the thalamus (42). The pathological discharge patterns can be replaced by HF spiking or suppressed depending on whether the efferent fibers of the stimulated nucleus are excitatory or inhibitory, respectively (41, 44, 45). Several contributing mechanisms, resulting in the observed effects of HF DBS might be membrane inhibition, jamming, excitation of excitatory and inhibitory afferents, excitation of efferents and plasticity (46). In particular, HF stimulation of afferent axons projecting to STN can account for therapeutic effects of HF DBS (21). The stimulation-induced effects are considered as a combination of local and non-local impacts of the stimulation, where HF DBS seems to strongly alter the neuronal firing and modulate the pathological neuronal activity.

KEY CONCEPT 1. Deep brain stimulation (DBS) Standard therapy for medically refractory movements disorders, e.g., Parkinson’s disease and essential tremor. It requires a surgical treatment, where depth electrodes are chronically implanted in target areas like the thalamic ventralis intermedius nucleus or the subthalamic nucleus. For standard DBS electrical high-frequency (>100 Hz) charge-balanced pulses are permanently delivered via depth electrodes.

Human data demonstrate that HF DBS of the STN can suppress the pathological neuronal activity in the β band, which correlates with induced improvement of PD symptoms (47–50), whereas low-frequency stimulation at around 20 Hz enhances synchronization at similar frequencies. A modeling study (51) suggested that the HF periodic DBS may induce a chaotic desynchronization, while a desynchronizing impact of a periodic forcing on synchronized populations seems to be a rather general phenomenon (52). The effect of HF DBS may, however, depend on whether excitatory or inhibitory structures are stimulated (53). PD-related oscillatory activity suppressed during HF stimulation reemerges after cessation of the stimulation within a few tens of seconds (36, 48, 49). Other studies did not, however, observe any marked attenuation of the β-band power after STN DBS has been discontinued (54, 55). Analogously, the therapeutic effects of HF DBS on PD symptoms also quickly disappear after stimulation cessation (56). These observations reflect the fact that standard HF DBS has neither long-lasting electrophysiological nor clinical aftereffects, and the HF DBS therapy can be effective only when the neuronal target structures are permanently stimulated. In 309 PD patients (57), it was shown that best motor outcome of STN DBS was achieved with stimulation contacts localized within the STN as compared with the zona incerta. Accordingly, not just fibers, but neuronal populations appear to be optimal target for DBS. In this context, it might be interesting that in a computational study in isolated oscillatory STN neurons HF DBS suppresses the neuronal spiking, where the results obtained in this rather simple model resemble the clinically observed relation between stimulation amplitude and stimulation frequency required for therapeutic efficacy (58). However, in spite of many beneficial effects, in some patients DBS may not help, or may cause side effects, or the therapeutic effects may wear off over time (59–62).

KEY CONCEPT 2. Desynchronization A process inverse to synchronization, where initially synchronized oscillating systems desynchronize as parameters change or they do so under the influence of appropriate external stimulation. Desynchronization is important, for example, in neuroscience and medicine, were pathologically strong synchronization of neurons may severely impair brain function as, e.g., in Parkinson’s disease or epilepsy.

KEY CONCEPT 3. Long-lasting stimulation aftereffects The impact of the stimulation on the neuronal population, which essentially outlasts the stimulation cessation. Apart from acute therapeutic effects, i.e., effects occurring during stimulation, a significant suppression of symptoms and abnormal neuronal dynamics is preserved for a long post-stimulation time after the stimulation is completely switched off.

With the objective of finding more effective stimulation techniques, which specifically counteract an abnormal neuronal synchronization, a model-based development of novel stimulation methods has been initiated (63). Accordingly, synchronization control became a focus of the research nowadays, and several desynchronizing methods have been developed with the methods of non-linear dynamics and statistical physics (64–77). Some of these methods are based on closed-loop (delayed) feedback techniques, where the mean field of the neuronal ensemble is measured, preprocessed, and fed back as a stimulation signal, which desynchronizes the stimulated neurons in an intrinsic demand-controlled way (68–70, 72–74, 76). Other methods utilize phase-resetting techniques (64–67).

In computational models used for developing and optimizing novel stimulation techniques, one should take into account spike timing-dependent plasticity (STDP) (78–85), a fundamental property of neuronal tissue, according to which synaptic weights depend on the neuronal firing pattern. It was shown experimentally that synaptic plasticity enhances neuronal synchronization (86). From the kindling phenomenon, in the context of epilepsy (87), where preparatory stimulation induces the spontaneous production of epileptic seizures, it is well known that neural networks may learn pathologically strong synaptic interactions (88, 89). The novel desynchronizing stimulation protocols are designed to invert this pathological process via anti-kindling (90). The very goal is to stimulate in a way that the formerly affected neuronal populations unlearn their pathological connectivity and, hence, their tendency to produce pathological synchronization, where the physiological neuronal activity is re-established on a long-term basis (53, 90–95). Put otherwise, the stimulation aims at inducing long-lasting therapeutic effects, which outlast the cessation of stimulation.

KEY CONCEPT 4. Spike timing-dependent plasticity (STDP) A fundamental property of the nervous system, where neurons continuously regulate the strength of their synaptic connections in relation to the mutual timing properties of their firing or bursting.

KEY CONCEPT 5. Anti-kindling A process inverse to the kindling phenomenon know in the context of epilepsy, where neural networks may learn pathologically strong interactions and spontaneously produce epileptic seizures. Anti-kindling manifests itself when due to desynchronizing stimulation neuronal populations unlearn their pathological connectivity, and physiological neuronal activity is re-established on a long-term basis.

So far, several theoretical predictions concerning the anti-kindling properties of one of the novel desynchronizing techniques, coordinated reset (CR) neuromodulation (53, 66, 67, 90–93), have been verified experimentally. The mechanism of CR neuromodulation is based on the phase reset of oscillatory neuronal activity (66, 67). This technique appears to be effective under a variety of conditions and stimulation modalities since the phase reset is a universal phenomenon. A phase reset of rhythmically active neurons can be achieved, e.g., by hyperpolarizing or depolarizing electrical pulses (96–99), excitatory, or inhibitory post-synaptic potentials (100–104), sensory stimulation (105–108), and transcranial magnetic stimulation (109). The resetting impact and the induced transient desynchronization of an electrical short-pulse stimulation, on which the CR technique is based, have been reported *in vivo* for coupled neuronal bursters in the paddle fish (99). Long-lasting desynchronizing effects of CR stimulation have been investigated in detail in theoretical studies (53, 90, 91), and the results have experimentally been confirmed *in vitro* in epileptic rat hippocampal slice (110). The beneficial therapeutic long-lasting aftereffects of electrical CR stimulation at weak intensity have been observed in the 1-methyl-4-phenyl-1,2,3,6-tetrahydropyridine (MPTP)-treated macaque monkeys, in contrast to CR stimulation at high intensity and to standard HF DBS (111). It was shown that unilateral weak-intensity CR stimulation delivered to the STN of parkinsonian MPTP monkeys for only 2 h per day during 5 consecutive days leads to significant and sustained therapeutic aftereffects for at least 30 days, while standard 130 Hz DBS and strong-intensity CR stimulation have no or only little aftereffects, respectively (111). Lasting aftereffects of electrical CR stimulation of the STN were also observed in Parkinson patients stimulated during three consecutive days in two daily sessions of up to 2 h (112).

KEY CONCEPT 6. Coordinated reset (CR) neuromodulation An effectively desynchronizing control technique, where a population of synchronized neurons is stimulated via several stimulation sites in such a way that spatially and timely coordinated phase resets are achieved in subpopulations assigned to each of the stimulation sites. This method was suggested for counteraction of abnormal neuronal synchronization characteristic for several neurological diseases. CR-induced long-lasting desynchronization has been verified in pre-clinical and clinical studies.

As discussed above, the effects of HF DBS may be quite different, depending on the target, e.g., neuronal populations as opposed to fibers, being stimulated. In contrast, computational studies showed that CR stimulation effectively induces anti-kindling by direct somatic stimulation as well as by excitatory or inhibitory synaptically mediated stimulation (94, 95). The latter stimulation setup might correspond to stimulation of afferent or efferent fibers, or sensory stimulation, where the stimulation signals arrive at the neural target population as post-synaptic potentials. Acoustic CR stimulation has been suggested for desynchronizing the neural synchrony underlying tinnitus (94) and successively verified in a clinical proof-of-concept study in tinnitus patients treated with non-invasive acoustic CR stimulation (26, 27, 29). It turned out that acoustic CR stimulation can significantly counteract both tinnitus symptoms and the underlying pathological neuronal synchronization in a tinnitus-related network of brain areas together with normalization of effective connectivity between these brain areas (26, 27, 29). Below we illustrate the mechanism and properties of CR neuromodulation in more detail and compare it to noise stimulation in neuronal networks with STDP.

## 2 STDP-Induced Multistability

**Figure 1:**
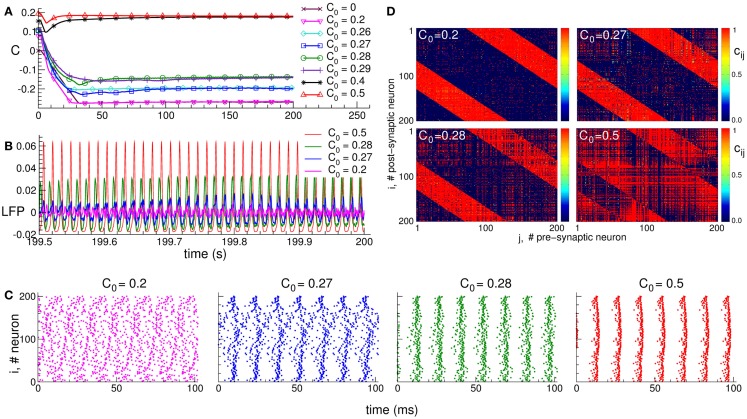
**Plasticity-induced multistability of synchronized and desynchronized states in an ensemble of Hodgkin–Huxley neurons (113, 114) with STDP**. **(A)** Time courses of the mean synaptic weights $C\left(t\right)=N^{-2}\sum_{i,j}\mathrm{sign}\left(M_{\mathrm{ij}}\right)c_{\mathrm{ij}}\left(t\right)$, where *N* is the number of neurons, *c_ij_*(*t*) is the individual synaptic weight from pre-synaptic neuron *j* to post-synaptic neuron *i*, and $M_{\mathrm{ij}}=\left(1-d_{\mathrm{ij}}^{2}∕\sigma_{1}^{2}\right)\exp\left(-d_{\mathrm{ij}}^{2}∕2\sigma_{2}^{2}\right)$ is the spatial coupling profile in the form of a Mexican hat with distance *d_ij_* between neurons, and parameters σ_1_ and σ_2_. The positive (negative) value of *M_ij_* indicates an excitatory (inhibitory) interaction, which corresponds to closely (distantly) located neurons. Boundary conditions are periodic. Initial synaptic weights {*c_ij_*(0)} are randomly Gaussian distributed around the mean value *C*_0_ (indicated in the legend) with SD 0.01. **(B)** Time courses of the DC-balanced local field potential $\mathrm{LFP}\left(t\right)=N^{-1}\sum_{j=1}^{N}s_{j}\left(t\right)$, where *s_j_*(*t*) is the post-synaptic potential of neuron *j*, of four stable synchronized and desynchronized regimes observed for the initial coupling matrices from **(A)** with mean values *C*_0_ indicated in the legend. **(C)** The corresponding raster plots of the neuronal spikes for the four stable states mentioned above. The initial mean values *C*_0_ are indicated in the plots. **(D)** The corresponding coupling matrices developed in the neuronal ensemble due to STDP for these four stable states. The initial mean values *C*_0_ are indicated in the plots. The oblique red stripes visible in the plots are the inhibitory connections, whereas the other connections are excitatory according to the considered Mexican hat coupling profile. The plots are adapted from Ref. (95), where further details can be found.

It is well known that synapses in neuronal networks are involved in adaptation processes, where their efficacy is governed by spike timing-dependent plasticity (STDP), and the synaptic weights are either potentiated or depressed depending on the order of the spiking times of pre- and post-synaptic neurons (78–85). Adaptive synapses greatly extend the complexity and richness of the neuronal dynamics and functions of neuronal networks. STDP can, for instance, induce an overwhelming multistability of neuronal dynamical regimes (90, 95, 115, 116). Depending on the initial conditions, the system may converge to one or another of many coexisting stable states, which are characterized by different synaptic connectivities and extents of synchronization among neurons. This phenomenon is illustrated in Figure 1. The mean coupling *C*(*t*) of the neuronal population saturates at different limit states depending on the initial distribution of the time-dependent synaptic weights *c_ij_*(*t*), which define the coupling strength from pre-synaptic neuron *j* to post-synaptic neuron *i* (Figure 1A). The neuronal population with STDP may thus exhibit diverse stable regimes of different connectivity strength. In these regimes, the collective dynamics of the neurons is also different. In particular, the oscillations of the local field potential (LFP) differ by their amplitude, such that stronger coupling leads to more pronounced LFP oscillations (Figure 1B). The LFP amplitude reflects the amount of synchronization among neurons, and it increases if the neurons self-organize in a synchronized rhythmic firing (Figure 1C). The coupling strength and neuronal synchronization are in correspondence with each other: stronger coupling results in stronger synchronization.

KEY CONCEPT 7. Multistability Co-existence of two (in the case of bistability) or more different stable regimes in the state space of a system for the same values of system parameters. These states can be realized for different initial conditions or under perturbations shifting the system state to the corresponding regime. In biological systems, multistability is a common phenomenon.

The dependence of the final coupling regimes on the initial connectivity is based on the above interrelation between the coupling strength and neuronal synchronization. Strong initial coupling synchronizes neurons from the very beginning. The synchronized neuronal dynamics then influences the synaptic weights via STDP, and the latter get even more potentiated, if the neurons are synchronized strongly enough with narrowly distributed relative spike timing (90, 95). This, in turn, enhances the neuronal synchronization. Such a self-organization process eventually results in a stable strongly coupled and synchronized regime. On the other hand, if the initial coupling and, thus, neuronal synchronization are weak, the synaptic weights will be depressed, and a stable weakly coupled and desynchronized regime will be established in the neuronal population due to STDP. Along with the above two limiting regimes, many other regimes of intermediate coupling strength and synchronization may coexist (Figure 1).

The structure of the synaptic connectivity also varies when the STDP-induced regimes of strong or weak coupling are realized in the neuronal population. In the weakly coupled and weakly synchronized regime, for instance, the excitatory connections are suppressed, whereas the inhibitory connections are potentiated (Figure 1D for *C*_0_ = 0.2). For other coupling regimes established in the neuronal ensemble due to STDP, an increase of the excitation is accompanied by a simultaneous decrease of the inhibition among neurons (Figure 1D). This relation is reflected by the extent of synchronization in the neuronal population, such that the synaptic connectivity dominated by excitatory connections leads to strongly synchronized neurons. On the other hand, enhanced inhibitory interactions with suppressed excitatory connections lead to a weakly synchronized neuronal activity.

The discussed relation between the connectivity and collective dynamics of the neuronal ensembles with adaptive synapses opens an approach for the control of neuronal synchronization. The STDP-induced multistability plays an important role to this. Indeed, by an appropriate stimulation the neuronal ensemble can be shifted from abnormally synchronized regime to a weakly coupled and desynchronized regime, which stably coexist with each other. For this, the neurons have to be stimulated in such a way that they approach a weakly synchronized regime, i.e., the stimulated neurons get desynchronized. Since the synaptic connectivity is influenced by the neuronal activity via STDP, the desynchronizing neuronal firing will depress the excitatory synapses. Accordingly, a desynchronizing stimulation will also normalize the connectivity such that an anti-kindling process will be initiated: the stimulated neuronal population will be shifted from a strongly coupled and synchronized regime to a stable weakly coupled and desynchronized regime. The latter regime persists after stimulation cessation, which manifests the long-lasting effect of a desynchronizing stimulation in neuronal networks with STDP. Below we illustrate this approach for two stimulation techniques: CR neuromodulation and stimulation by independent noise.

## 3 Anti-Kindling by Coordinated Reset Neuromodulation

**Figure 2:**
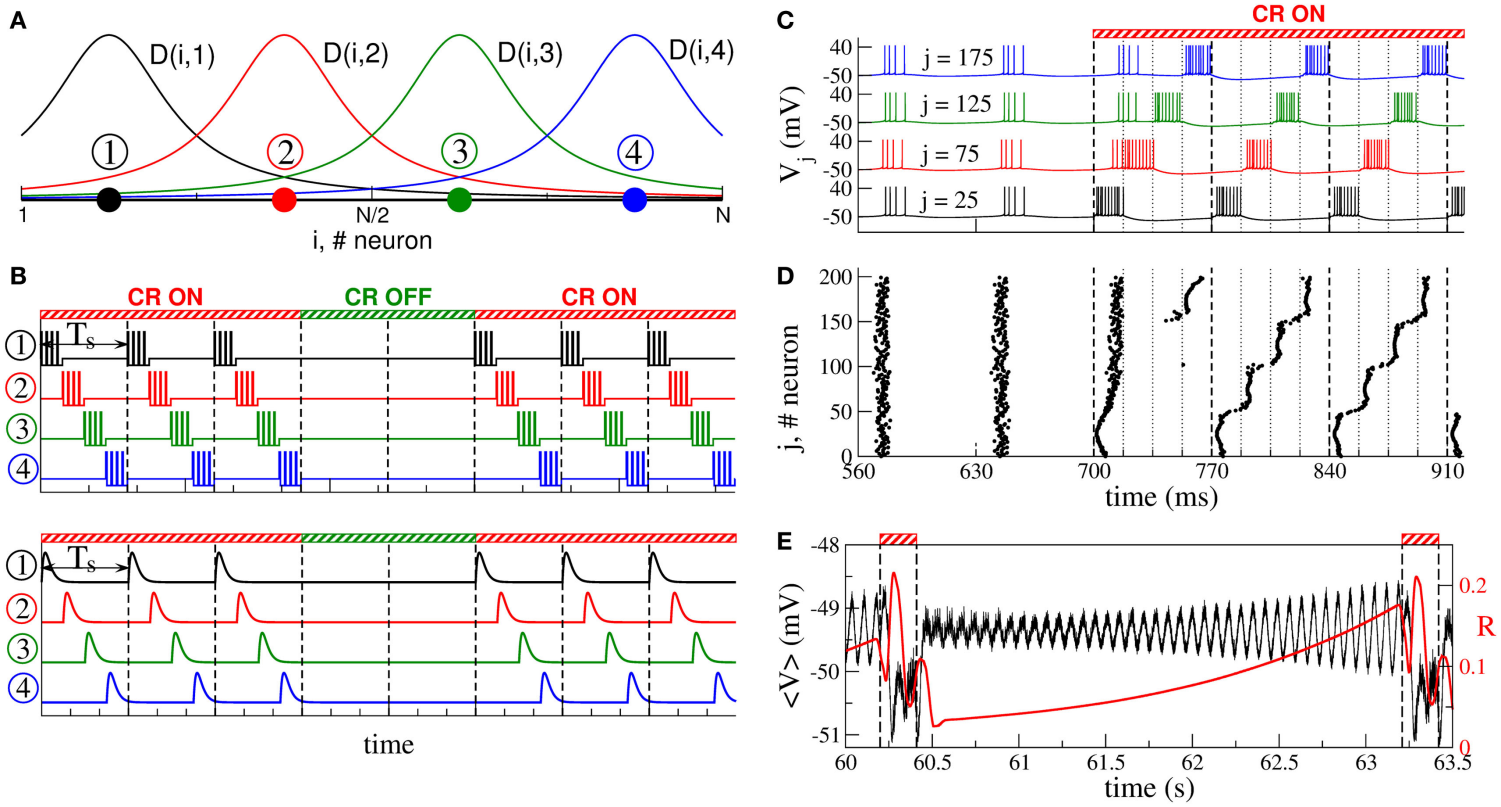
**Stimulation setup of CR neuromodulation and its impact on synchronized neurons**. **(A)** Schematic localization of four stimulation sites (filled circles) within the neuronal population and the corresponding spatial profiles (solid curves) of current decay in the neuronal tissue with the distance from the stimulation site. **(B)** Stimulation signals of electrical CR neuromodulation composed of short-pulse trains of charge-balanced pulses (upper plot), and stimulation signals of synaptically mediated, e.g., sensory CR neuromodulation in the form of post-synaptic potentials (lower plot). Vertical dashed lines separate the stimulation cycles of length *T_s_*, where each stimulation site is activated once. **(C)** Time courses of the membrane potentials *V_j_* of four neurons selected from the ensemble of coupled adaptive exponential integrate-and-fire (aEIF or AdEx) bursting neurons (117–119) without STDP stimulated by CR neuromodulation. The neurons are assigned to each of the four stimulation sites from plot **(A)** located at the same lattice coordinates (indices *j* are indicated in the plot). Stimulation begins at *t* = 700 ms and shifts the bursting of the neurons with respect to each other. The vertical dashed lines comprise the CR stimulation cycles of length *T_s_* = 70 ms, and the dotted lines indicate the time intervals, where the corresponding stimulation site is active. **(D)** The corresponding raster plot of the burst onsets of all *N* = 200 neurons. **(E)** Time courses of the ensemble mean field of AdEx neurons $\left\langle V\right\rangle =N^{-1}\sum_{j=1}^{N}V_{j}$ (black curve) and the order parameter $R=|N^{-1}\sum_{j=1}^{N}e^{i\psi_{j}}|$ (120) (red curve, scale on the right vertical axis), where ψ*_j_* is the phase of neuron *j* (121), between two successive ON epochs (indicated by dashed vertical lines and bars on the top of the plot) of 3 stimulation cycles. The plots are adapted from Ref. (122), where further details can be found.

According to the CR neuromodulation algorithm (66, 67), the neuronal target population is stimulated via several stimulation sites, such that the entire population is divided into the same number of sub-populations, each of them receiving the stimulation mostly from one of the stimulation sites (Figure 2A). The stimulation signals for electrical CR stimulation are brief trains of high-frequency charge-balanced electrical pulses administered to the neuronal tissue via different stimulation sites (Figure 2B, upper plot). Electrical CR neuromodulation requires an electrode implantation into the neuronal target population or into fibers projecting onto a target population, as, for example, in the case of DBS (30). In fact, CR neuromodulation has initially been developed for the application to electrical DBS (66, 67). The stimulation signals can also have the form of post-synaptic potentials (Figure 2B, lower plot). The latter stimulation modality is realized when excitatory or inhibitory axons terminals or fibers are electrically stimulated, and the stimulation signals arrive at the neuronal target population as post-synaptic potentials evoked in the dendrites. Furthermore, such an indirect, synaptically mediated stimulation modality can model the impact of sensory, e.g., acoustic CR neuromodulation (29, 94).

During one stimulation cycle of length *T_s_*, the stimulation sites are sequentially activated and deliver stimulation signals to different sub-populations of the neuronal target ensemble (Figures 2A,B). The stimulation signals are administered via stimulation sites in a time-coordinated manner, such that the next stimulation site is activated with a delay of *T_s_*/*M* (*M* is the number of stimulation sites) after the activation of the preceding stimulation site. The stimulation period *T_s_* is optimally chosen close to the mean period of the synchronized neurons. Within one cycle of length *T_s_* each stimulation site is activated once. The stimulated neurons are sequentially reset by the corresponding stimulation signals such that, after a few stimulation cycles, the sub-populations assigned to different stimulation sites get phase shifted with respect to each other (Figures 2C,D). The total synchronization is thus replaced by a cluster state (66, 67, 122, 123). If the stimulation is then switched off, the neurons typically relax from the cluster state to a desynchronized state, which is characterized by low-amplitude oscillations of the mean field (Figure 2E), as discussed in Section 2. The stimulation-free neurons without STDP will eventually resynchronize if left unperturbed (Figure 2E). Hence, to maintain a desynchronized neuronal firing, CR stimuli have to be administered repetitively, where a few stimulation ON cycles are recurrently followed by a few stimulation OFF cycles (Figures 2B,E). In experimental and clinical studies, 3 ON cycles and 2 OFF cycles were used (Figure 2B) (29, 111, 112).

**Figure 3:**
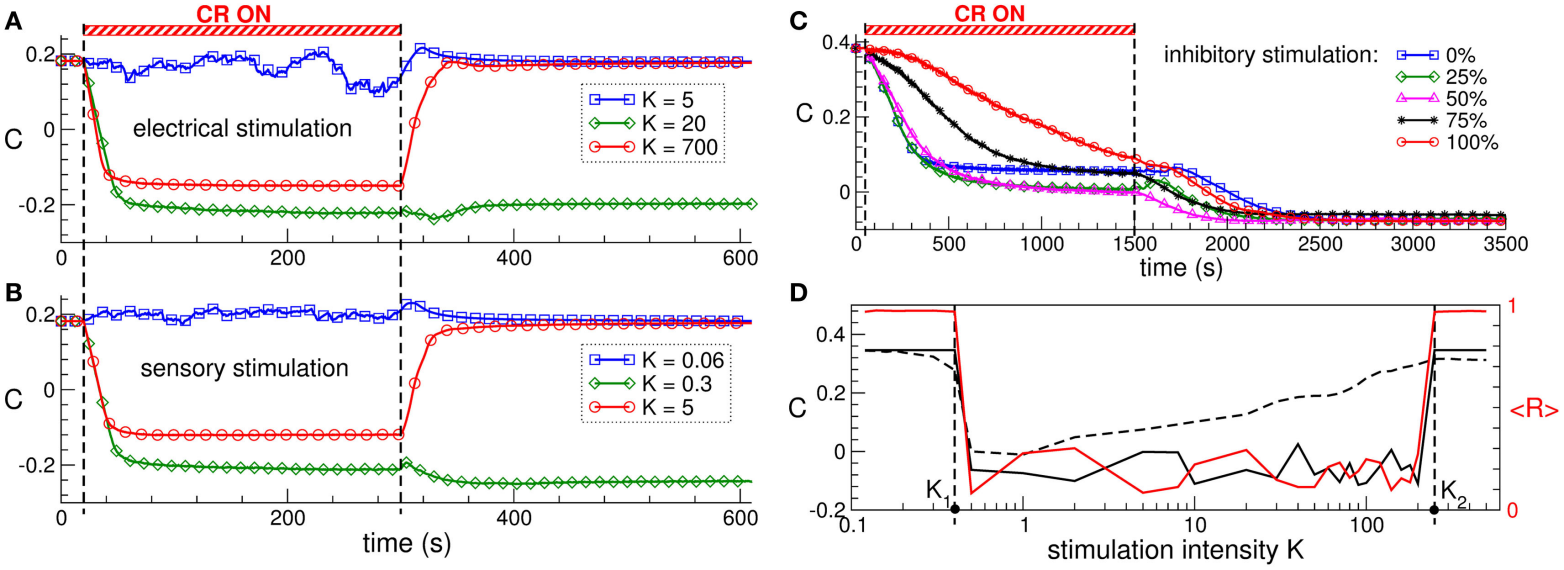
**Stimulation-induced anti-kindling of neuronal ensembles with STDP by electrical and sensory CR neuromodulation**. **(A,B)** Time courses of the mean synaptic weights *C*(*t*) (see Figure 1 for definition) of the ensemble of Hodgkin-Huxley neurons (113, 114) with STDP for stimulation intensities *K* indicated in the legends. The stimulation time interval is indicated by the red bar and by the vertical dashed lines for **(A)** direct electrical stimulation and **(B)** indirect, synaptically mediated, e.g., sensory excitatory stimulation administered to a strongly coupled and synchronized regime as in Figure 1 for *C*_0_ = 0.5. **(C)** Time courses of the mean synaptic weights *C*(*t*) of the ensemble of FitzHugh–Rinzel bursting neurons (124, 125) with STDP for different fractions of the neuronal population of randomly selected neurons receiving an inhibitory synaptically mediated CR stimulation as indicated in the legend. The rest of the neuronal ensemble receives an excitatory stimulation. The stimulation time interval is indicated by the red bar and by the vertical dashed lines. **(D)** Optimal range of the stimulation intensity for long-lasting effects of synaptically mediated inhibitory CR neuromodulation for FitzHugh–Rinzel bursting neurons with STDP. Dashed and solid black curves depict the mean synaptic weights *C_on_* and *C_off_* registered at the ends of CR stimulation epoch and post-stimulation transient, respectively. The red solid curve shows the order parameter <*R*(*t*)> time averaged over the last 3 s of the post-stimulation transient (the scale on the right vertical axis). Vertical dashed lines indicate the parameter interval *K*∈(*K*_1_, *K*_2_), where the inhibitory CR stimulation is effective in inducing an anti-kindling. The plots are adapted from Ref. (95), where further details can be found.

The direct electrical as well as the indirect, synaptically mediated CR neuromodulation can have a well-pronounced desynchronizing effect on the stimulated neuronal population. When administered to neuronal populations with STDP, CR neuromodulation does not only influence the collective dynamics but also the pattern of the synaptic couplings among the neurons, i.e., it may induce a rewiring of the stimulated neuronal ensemble: the control of the collective dynamics enables a control of the connectivity. Time courses of the mean synaptic weight *C*(*t*) before, during, and after direct electrical CR stimulation and excitatory synaptically mediated CR stimulation are shown in Figures 3A,B, respectively. Both stimulation modalities have very similar impact on strongly coupled and synchronized neurons. Weak stimulation only slightly perturbs the connectivity, and the neurons remain strongly coupled after stimulation offset (Figures 3A,B, blue curves). Optimal CR neuromodulation of intermediate intensity induces a long-lasting anti-kindling: synaptic connectivity is reshaped during the stimulation, and the stimulated neuronal population is shifted to a weakly coupled and, thus, desynchronized regime, which persists after stimulation is switched off (Figures 3A,B, green curves). Too strong CR neuromodulation, however, fails to induce such aftereffects, and only an acute (during stimulation) normalization of synaptic connectivity can be observed (Figures 3A,B, red curves).

Synaptically mediated CR neuromodulation is also effective in inducing anti-kindling if a fraction of neurons receives an inhibitory stimulation up to a fully inhibitory input (Figure 3C). Any of these stimulation modalities exhibits an optimal range of the stimulation intensity, where a long-lasting suppression of abnormal synaptic connectivity and neuronal synchronization takes place (Figure 3D). The emergence of the upper bound of the optimal parameter range is based on the non-linear mechanism of CR neuromodulation and indicates that CR neuromodulation is most effective for low stimulation intensities. This constitutes an essential difference to the standard HF DBS, where the stimulation strength has to be of sufficient strength in order to effectively suppress the PD symptoms, on the one hand, but the rise of adverse effects bounds its admissible range, on the other hand (17, 126–128). The efficacy of weak CR neuromodulation has been confirmed in a pre-clinical study of CR neuromodulation in the MPTP-treated parkinsonian monkeys (111). Sustained long-lasting aftereffects on motor function in MPTP monkeys can be induced by CR stimulation of the subthalamic nucleus (STN) at the stimulation intensity equal to a third of the stimulation strength necessary for an acute suppression of symptoms by the standard HF DBS. In contrast, delivering CR stimulation at a larger stimulation strength equal to that of HF DBS led to only weak and considerably shorter CR aftereffects (111).

The observed similarity of the stimulation efficacy in inducing anti-kindling by direct electrical CR stimulation and indirect, excitatory, or inhibitory synaptically mediated CR stimulation (Figure 3) provides a basis for a diversity of possible modalities for the control of pathological neuronal synchronization by CR neuromodulation. On the one hand, the local effects (realized by direct stimulation of the neurons’ somata or stimulation of afferent axon terminals) and the non-local effects (realized by stimulation of efferent fibers) of deep brain CR stimulation may be similar or even identical. This is in contrast to the stimulation effects of the standard HF DBS, where local and non-local effects may differ considerably (33). CR neuromodulation can thus normalize the neuronal activity not only in the directly stimulated neuronal population but also in the structures downstream, which might significantly extend the spectrum of possible targets structures for brain stimulation. On the other hand, a basic prerequisite for sensory CR stimulation is fulfilled, where CR stimulation is shown to be effective for stimulation with excitatory or inhibitory post-synaptic potentials, which models an indirect stimulation of the neuronal target population by sensory input. Hence, the DBS-oriented concept of CR-induced desynchronization (66, 67) and anti-kindling (90) might be extended to a more general CR neuromodulation concept, which can be realized by invasive as well as non-invasive, sensory (e.g., acoustic) stimulation. Sensory CR stimulation has been suggested and successfully applied for counteraction of tinnitus symptoms and normalization of the effective connectivity and functional patterns of activity in tinnitus patients (26, 27, 29, 94).

## 4 Self-Organized Noise Resistance

**Figure 4:**
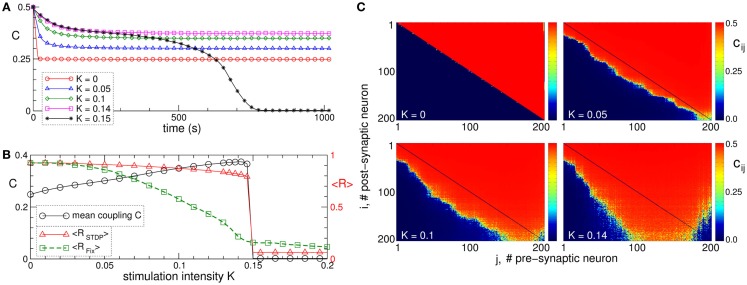
**Constructive effect of independent random input on the synaptic weights and self-organized noise resistance of the ensemble of Hodgkin–Huxley neurons (113, 114) with STDP**. **(A)** Time courses of the mean synaptic weight *C*(*t*) for different intensities of the random stimulation as indicated in the legend, where each neuron receives an independent random excitatory synaptic input. The initial synaptic weights *c_ij_*(0) are randomly Gaussian distributed around the mean value *C*_0_ = 0.5 with SD 0.02. **(B)** Mean synaptic weight *C*(*t*) (scale on the left vertical axis) and the time-averaged order parameters <*R_STDP_*> and <*R_Fix_*> (scale on the right vertical axis) for the ensemble with and without STDP, respectively, versus input intensity *K*. For the network without STDP, the coupling matrix is fixed as for the stimulation-free case (see plot C for *K* = 0). **(C)** Coupling matrices established in the neuronal ensemble due to STDP for the input intensities indicated in the plots. For illustration, the neurons are sorted with respect to increasing natural spiking frequency. The plots are adapted from Ref. (129), where further details can be found.

Synchronization in networks of coupled neurons can be destroyed by an independent random input, which is known to be a powerful method for counteracting synchronization in coupled oscillators without STDP (130). In neuronal networks with adaptive synapses, the situation becomes more complicated. In fact, a random noise stimulation administered to a population of strongly coupled and synchronized neurons with STDP may enhance the amount of synaptic coupling among the neurons (Figure 4A) (129). Stimulation-free neurons get strongly coupled, and a unidirectional (coupling only in one direction between two neurons) hierarchical coupling topology is established due to STDP for strong enough initial coupling (Figures 4A–C for *K* = 0) (129, 131, 132). If a random independent input is applied to the neuronal ensemble with STDP, the amount of coupling in the stimulated ensemble is significantly enhanced (Figure 4A). The random perturbations have a constructive effect on the dynamics of the synaptic weights, and the noise promotes the development of bidirectional (coupling in both directions between two neurons) synaptic connections among neurons (Figure 4C).

The mean coupling increases as the input strength grows and demonstrates a resonance-like behavior, where there exists an optimal noise intensity, resulting in a maximal coupling among neurons (Figure 4B, black curve). The neurons counteract the desynchronizing effect of the noise by reorganizing their synaptic connectivity and increasing its strength, such that the amount of synchronization in the neural ensemble with STDP is relatively well preserved for moderately strong noise (Figure 4B, red curve). On the other hand, synchronization is strongly suppressed by the noise stimulation in the same neuronal network for fixed connectivity (Figure 4B, green curve). This mechanism constitutes a self-organized resistance to independent noise in the neuronal networks with STDP, where the latter preserves the existing level of neural synchrony by a homeostatic regulation of the synaptic connectivity in response to external noise perturbations. Therefore, an independent noise can by no means be considered as an effective method for desynchronization of oscillatory neural networks with STDP. Also, from the clinical standpoint these results may be important. In fact, they may contribute to a deeper understanding of why maskers and noisers show limited efficacy in counteracting tinnitus (133), the latter being associated with abnormal neural synchrony (10, 29).

## 5 Discussion

Computational, pre-clinical, and clinical proof-of-concept studies revealed that CR neuromodulation is effective in inducing a long-lasting desynchronization for a number of stimulation setups and demonstrates a great applicability (26, 27, 29, 53, 90, 91, 94, 95, 110–112). In particular, the computationally revealed fact that CR stimulation is effective, no matter whether it is directly delivered to the neurons’ somata or indirectly, via excitatory and/or inhibitory synapses (94, 95) may turn out to be crucial for several reasons:

- The effects of CR DBS might be considerably more robust with respect to variations of target structures as opposed to HF DBS. As discussed above, the effects of HF DBS strongly depend on the target.
- CR-induced desynchronization and anti-kindling might be achieved by means of a variety of invasive as well as non-invasive stimulation modalities, e.g., by DBS (111, 112), by acoustic (26, 27, 29), and by other types of sensory stimulation as well as by spinal cord stimulation (134, 135), e.g., to counteract abnormal neuronal synchrony underlying neurogenic pain (136–138).

CR neuromodulation has also been suggested to counteract cerebral hypo-activity found in a number of diseases including Alzheimer’s disease, schizophrenia, major depression, and bipolar affective disorders (139–143). A simple periodic stimulation may activate the stimulated neurons, but can also induce an undesirable enhancement of neuronal synchronization, significantly deteriorate symptoms, and even lead to kindling processes and evoke epileptiform and other types of abnormal activity (18, 20, 47, 89, 110). As computationally shown, CR neuromodulation can specifically counteract neuronal hypo-activity in a safe manner without promoting pathological synchronization by a multi-frequency and phase-shifted activation of the stimulated neuronal networks (144).

In the open-loop stimulation protocol of CR neuromodulation discussed above, the repetitive stimulus administration, after initial parameter calibration, is organized regardless of the state of the stimulated ensemble. CR neuromodulation can also be utilized with closed-loop stimulation protocol in a demand-controlled way, where parameters and timing of the stimulation can be adapted to the ongoing neuronal activity (66, 67). Here, two strategies can, for instance, be suggested (*i*) demand-controlled timing of the administration of identical stimuli, where the same stimulus is administered whenever the population tends to resynchronize, or (*ii*) demand-controlled length of administered pulse trains, where the length of the pulse trains is longer for stronger synchronization measured at the onset of the stimulation, see in Ref. (66, 67) for details. Note, closed-loop stimulation is naturally realized by feedback methods (68–70, 72–74, 76), which can be considered as further candidates for control of abnormal neuronal synchronization.

The closed-loop approach has already been tested for standard HF DBS under acute conditions in MPTP-monkeys (145) and PD patients (146). To this end, a short train (comprising 7 pulses at 130 Hz) was delivered through a pair of electrodes located in the globus pallidum interior (GPi) at a predetermined, fixed latency (80 ms) following each action potential recorded through an electrode placed in the primary motor cortex (M1) (145). This type of stimulation caused a stronger decrease of the firing rate of pallidal neurons together with a more pronounced decrease of the oscillatory neuronal activity along with a better amelioration of the MPTP-induced akinesia as compared to the standard continuous 130 Hz DBS. After cessation of this type of closed-loop DBS the initial firing pattern reverted back to the pre-stimulus levels (145). Another study (146) confirmed the efficacy of the closed-loop adaptive DBS (aDBS) in PD patients, where the onsets and offsets of HF stimulation were triggered by a threshold crossing by LFP in the β-band measured via the same stimulation electrode implanted in STN. For the same stimulation intensity and stimulation frequency (130 Hz), the aDBS can be about 30% more effective than standard continuous HF DBS, while <50% of the total electrical energy is delivered in the aDBS mode as compared to continuous HF DBS. Moreover, despite of the used fixed beta threshold, the triggered stimulation duration (per 10-s block) progressively drops over time during stimulation in the aDBS mode, which suggests that aDBS might lead to positive adaptive effects in pathological parkinsonian networks (146).

We have included computational data obtained in neuronal networks with STDP stimulated by independent noise for two reasons. First, these findings demonstrate how important it is to include STDP in neuronal networks in order to design and optimize desynchronizing stimulation techniques. Second, these findings illustrate how important the choice of the right stimulation technique is. Stimulation techniques that might intuitively be expected to have beneficial effects may actually have surprisingly counter-productive effects.

In several computational studies, parameters of CR neuromodulation have been optimized in model neuronal networks of different complexity with and without STDP (66, 67, 90, 95, 122, 123, 147). In particular, the impact of stimulation strength, number of stimulation sites, and stimulation timing was investigated in detail and related to the properties of the stimulated neuronal tissue. The obtained theoretical and experimental results indicate the robustness and broad applicability of CR neuromodulation, which may finally become a novel concept for the treatment of neurological disorders characterized by abnormal neuronal synchronization.

## Conflict of Interest Statement

Dr. Peter A. Tass had a contractual relationship with ANM Adaptive Neuromodulation GmbH. No financial interests exist related to the presented results. Dr. Oleksandr V. Popovych declares that the research was conducted in the absence of any commercial or financial relationships that could be construed as a potential conflict of interest.
